# A New Coracoclavicular Guider for Minimally Invasive Anatomic Coracoclavicular Reconstruction with Two TightRope Systems in Acute Acromioclavicular Joint Dislocation

**DOI:** 10.1038/s41598-019-51119-7

**Published:** 2019-10-08

**Authors:** Yi Zhao, Lei Tan, Wan Tang, Tiecheng Yu

**Affiliations:** 1 Department of Orthopedics, First Hospital of Jilin University, Changchun, 130021 China; 2 Department of Operating Theatre, First Hospital of Jilin University, Changchun, 130021 China

**Keywords:** Ligaments, Trauma

## Abstract

High-grade injuries of complete acromioclavicular (AC) joint disruption (types IV - VI) are typically treated surgically. Since the coracoclavicular (CC) ligament is most often used for stabilizing the AC joint, most reconstruction techniques to treat dislocation of this joint rely upon CC interval fixation. A TightRope system is usually used to augment the CC ligament to treat acute AC dislocations with arthroscopic assistance. The conventional arthroscopic technique employing one TightRope system is associated with some complications, including anterior subluxation of the clavicle and clavicular bony avulsion as a consequence of rotational movements. As an alternative, two TightRope systems can be used to anatomically reconstruct the CC ligament to avoid these complications. We present a new CC guider with which the surgeon can replicate the native CC ligament complex orientation using two TightRope systems via two minimally invasive incisions without arthroscopic assistance. This procedure relies upon the accommodation and stable placement of the clavicle and coracoid bone tunnels for the two TightRope systems in place of the trapezoid and conoid of the CC ligament. We retrospectively reviewed the outcomes for 16 patients with acute dislocation of the AC joint that had been treated by a single surgeon using a double-button fixation system. An independent reviewer conducted functional testing of these patients, including the use of Disability of Arm, Shoulder and Hand (DASH), Constant and visual analog scale (VAS) scores. Standard radiographs were used for assessing the CC distance for the impacted shoulder relative to that of the unaffected contralateral shoulderThe new CC guider leads to an excellent cosmetic result. Our clinical results show that this technique can be easily performed and is similarly invasive to other current arthroscopic techniques.

## Introduction

Acromioclavicular (AC) joint dislocations are classified as types I–VI based on radiographic findings using the Rockwood criteria^1^. Surgical treatment of type III injuries remains controversial, but such treatment is routine for type IV–VI injuries owing to their severe instability.

Coracoclavicular (CC) ligaments play a very important role in maintaining stability of the AC joint^2^. Therefore, CC interval fixation was recently used to surgically treat dislocation of the acromioclavicular (AC) joint^3^.

The TightRope system has recently been used to augment the CC ligament to treat acute AC dislocations with arthroscopic assistance^1^. Arthroscopic CC ligament reconstruction with one TightRope system (Arthrex, Naples, FL) is a new surgical technique for treating acute AC dislocations^4,5^. This system is an adjustable-loop length suspensory fixation device that was originally designed for use with arthroscopes^6^. The TightRope system is comprise of a pair of metal buttons, with one being ovoid and the other circular in shape. A loop No. 5 FiberWire (Arthrex) joins these together^6^. Under arthroscopy, one button of the TightRope system is engaged under the coracoid process through the coracoid tunnel, while the other is over the clavicle in between CC ligament insertion sites^7^. Tension on the No. 5 FiberWire is then used to maintain the CC interval^8^.

In evaluating clinical outcomes, CC fixation with one TightRope system for acute AC dislocation has led to satisfactory recovery of shoulder function^1^. However, the TightRope-system fixation technique results in several new potential complications, such as recurrent dislocation, bony clavicle erosion, and coracoid process pull-through^1^. One reason for CC interval reduction failure is that one TightRope system cannot restore native CC ligament anatomy^9^. As the native CC ligaments have distinct anatomical attachments from this system, this results in altered AC joint stability and functionality^10,11^.

The CC ligament complex is composed of the conoid and trapezoid, with are primary shoulder suspensory ligaments^11,12^. Although structurally similar, these ligaments are different with respect to function and orientation, thereby mediating AC joint stability^12^. Ideal reconstruction methods should roughly replicate the trapezoid and the conoid of the CC ligament in order to ensure that a normal CC interval is maintained until the soft tissue around the CC ligaments fully heals^13^.

Therefore, we used two TightRope systems to anatomically reconstruct the CC ligament to achieve ideal fixation and avoid complications^13^.

**Figure 1:**
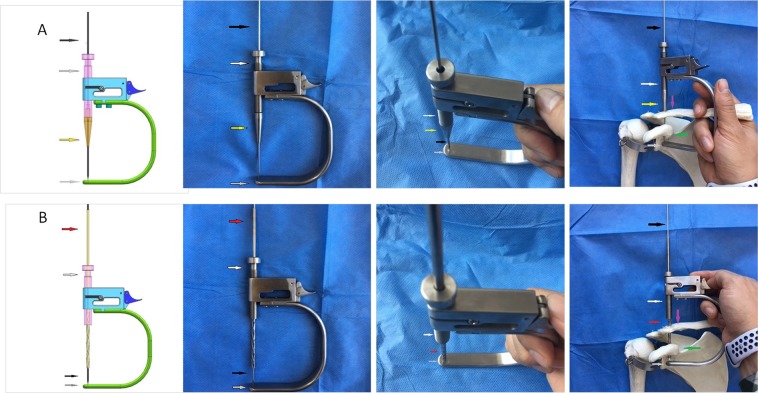
The new coracoclavicular (CC) guider. The new CC guider has two ends. (**A**) One end is the drill stop (dark arrow), which is placed under the coracoid basis (green arrow). The other end is placed on top of the clavicles (purple arrow), which consists of a drill sleeve (white arrow) and pin sleeve (yellow arrow). When the guide pin is introduced into the pin sleeve, the guider’s pin sleeve is centered on the guide pin (black arrow). (**B**) Once the guide pin’s position is ready, the guider’s pin sleeve is removed, and the guider’s drill sleeve is left *in situ*. Then, the performer inserts the 4-mm-diameter cannulated drill bit (white arrow) into the guider’s drill sleeve with the guide pin inside the cannulated drill bit, and it is advanced along the pin and through the clavicle and coracoids. The guider’s drill sleeve is centered on the drill bit (red arrow).

**Figure 2:**
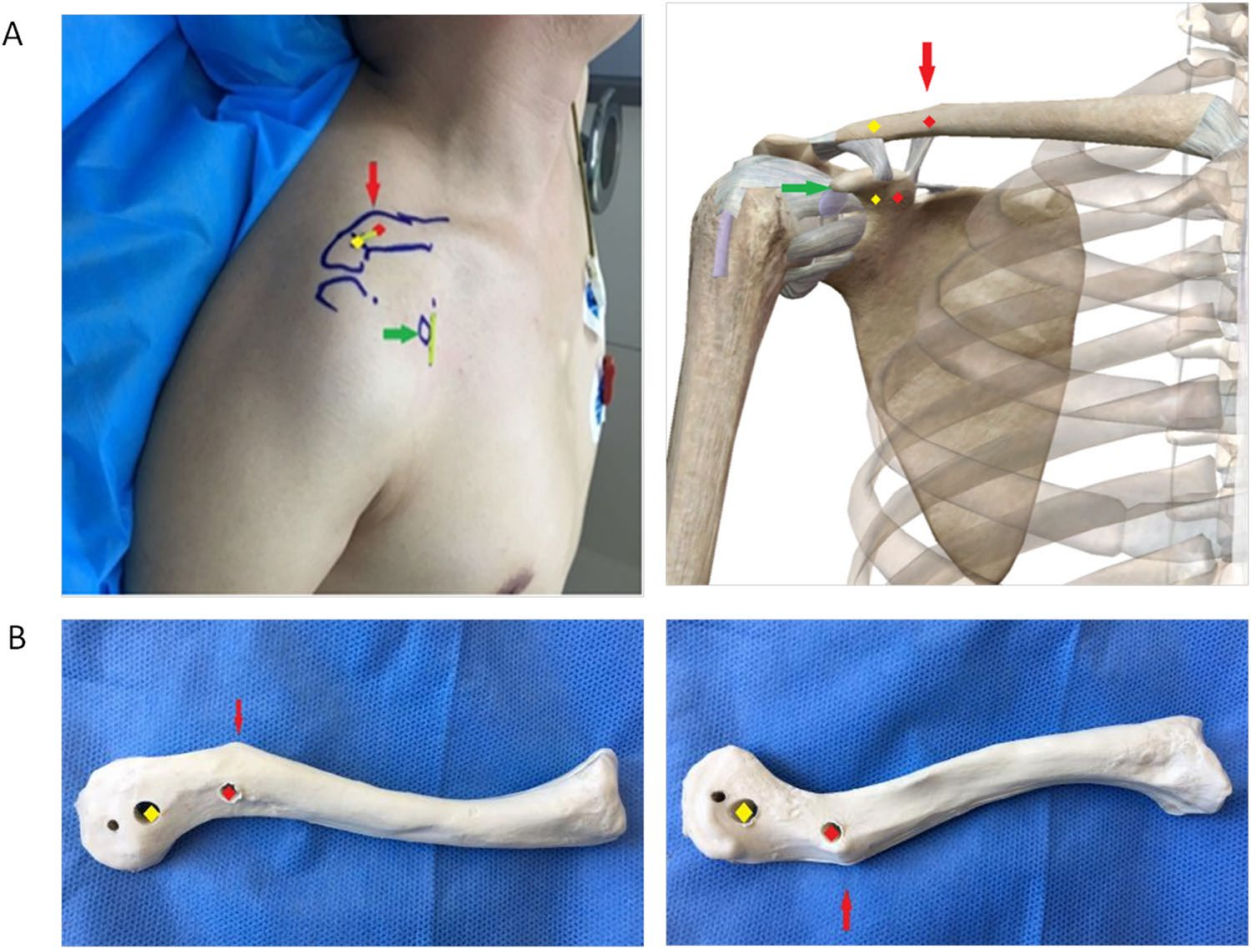
The conoid and trapezoid clavicular tunnel placements are marked on the top of the clavicle. **(A)** The distal end of the affected clavicle is palpated, and its outline is marked on the body surface. The coracoid is palpated and marked, which is indicated by the green arrow. The distal angle of the clavicle is indicated by the red arrow. Its base is the conoid tubercle, which is attached by the conoid ligament. (**B**) The center of this part of the clavicle is marked red to indicate conoid clavicular tunnel placement on top of the clavicle. The center from the red point to the distal end of the clavicle is marked yellow to indicate trapezoid clavicular tunnel placement on top of the clavicle.

In this paper, we present a novel CC guider (Fig. 1) with which we anatomically reconstructed CC ligaments via two minimal incisions with 2 TightRope systems (Fig. 2) without arthroscopic assistance. This technique benefits from being less invasive than arthroscopic procedures. Our clinical experience has shown that the CC guider provides a fast, simple, direct, reproducible, and minimally invasive means of achieving acute AC joint stabilization, in contrast to arthroscopic procedures.

## Patients and Methods

The guidelines for the care for human subjects of the First Hospital of Jilin University were observed at all times during this study, with patients providing written informed consent to participate and the Research Ethics Committee of the First Hospital of Jilin University having approved all study protocols.

Patients suffering traumatic AC joint dislocation that underwent surgical treatment using the two double-button fixation systems from January 2013 through July 2019 were enrolled in this study. Patient AC joint dislocation diagnoses were based on both clinical and radiographic assessment within a 2 week period following the original injury. The inclusion criteria were as follows: (1) acute AC joint dislocation ranked higher than grade III in the Rockwood classification system, (2) No prior shoulder injuries or surgeries, (3) no related injuries, and (4) a minimum of 12 months of follow-up. The same surgeon treated all patients.

**Figure 3:**
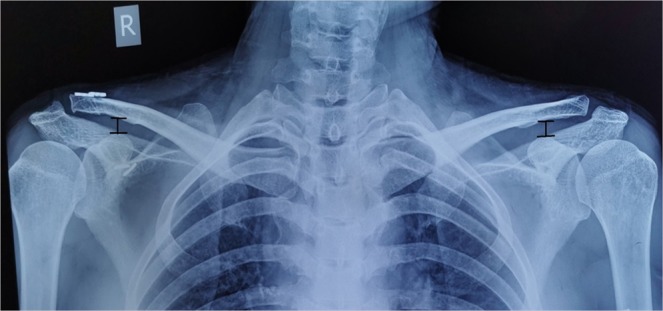
Radiograph illustrating the coracoclavicular distance. Radiograph illustrating the coracoclavicular distance as the vertical distance between the anterior–inferior border of the clavicle and the superior border of the coracoid process.

We followed 16 patients meeting these inclusion criteria (range: 12–30 months) (Table 1). The Disability of Arm, Shoulder and Hand (DASH) scoring system, Constant score, and the visual analog scale (VAS) scores were used to gauge shoulder function in these patients by an independent reviewer. The CC distance, defined as the distance from the clavicle anterior–inferior border and the coracoid process superior border, was determined based upon standard anteroposterior views of the AC arch in both preoperative images and those taken at final follow up for each shoulder (Fig. 3).

**Table 1:** Patients’ information.

No	Gender	Age	side	Dominant extremity	Rockwood classification of AC joint dislocation	Mechanism	Days (from injury to surgery)	Follow up (months)	VAS score		DASH score		Constant score		CC Distance (mm)		Return to work (weeks)	complication
									Pre-op.	Post-op.	Pre-op.	Post-op.	Pre-op.	Post-op.	Affected side	Contralateral side		
1	M	47	L	No	IV	CA	1	24	5	0	22	0	32	100	10.0	10.3	6	none
2	F	43	R	Yes	V	Fall	1	36	3	0	23	0	28	98	9.0	9.3	8	none
3	M	40	R	Yes	IV	CA	2	20	4	0	28	0	44	100	12.2	12	7	none
4	M	67	L	No	IV	fall	3	23	4	0	27	0	34	100	8.8	8.8	6	none
5	F	41	R	Yes	IV	CA	1	19	5	0	21	0	28	94	10.8	10.5	8	none
6	M	53	L	No	V	CA	2	33	4	0	23	0	39	90	11.6	11.5	8	none
7	M	45	L	No	IV	CA	1	20	4	0	24	0	30	100	11	11	7	none
8	F	49	R	Yes	IV	CA	2	17	5	0	18	0	26	100	8.3	8.3	6	none
9	M	52	L	No	V	fall	1	14	3	0	26	0	32	89	10	10	8	none
10	M	55	L	No	V	CA	1	18	4	0	28	0	41	98	9.6	9.3	6	none
11	M	40	L	No	V	CA	1	15	4	0	24	0	35	100	11.5	11.7	8	none
12	M	44	R	Yes	IV	CA	1	14	6	0	19	0	28	98	10.2	10.2	6	none
13	F	56	L	No	V	CA	1	19	3	0	15	0	32	88	9.5	9.4	8	none
14	M	59	L	No	V	fall	1	12	4	0	27	0	41	100	9.3	9.3	8	none
15	F	56	L	No	V	CA	2	14	3	0	26	0	27	100	10.3	10.8	8	none
16	F	58	L	No	V	CA	1	19	3	0	28	0	25	98	10	9.9	8	none

Note:

CA: Car accident.

VAS score: Visual Analogue Scale score.

DASH score: the Disability of Arm, Shoulder and Hand scoring system;

CC distance: the vertical distance between the anterior–inferior border of the clavicle and the superior border of the coracoid process.

## Surgical Technique

Patients with acute Rockwood IV–V injuries were surgically treated in this study. In individuals suffering from chronic AC joint separations, the possibility of full reduction of the clavicle must be established preoperatively. The patient was anesthetized before being placed in a supine position on a radiolucent operating table. His shoulders were padded with a 5-cm-thick cushion. One intravenous dose of cefazolin (1000 mg) was given prophylactically to all patients. A fluoroscopy unit with a large C-arm (to visualize the entirety of the clavicle from anteroposterior and apical oblique angles) was positioned on the contralateral side. This arm could be manipulated from the opposite side to simply imaging procedures during the operation as there was no need to step away from the surgical field.

After full preparation and draping of the entire clavicle and upper extremities the head was tilted away from the operative side by ~30° away from the operative side to increase the working area for the surgeon, while the drill bit was moved in a superior-to-inferior direction.

Two small incisions were made along Langer’s lines (Fig. 2A). First, the surgeon palpated the distal end of the affected clavicle and marked its outline on the body surface (Fig. 2A). The coracoid was also palpated and marked, which is indicated by the green arrow in Fig. 2A. The distal angle of the clavicle, could be easily identified, as indicated by red arrows (Fig. 2A,B). The base of the clavicle angle was the conoid tubercle, which was attached by the conoid ligament (Fig. 2A,B). The center of this part of the clavicle was marked for conoid clavicular tunnel placement on top of the clavicle, as indicated by red points (Fig. 2A,B). The center from the red point to the distal end of the clavicle was marked for trapezoid clavicular tunnel placement on top of the clavicle, as indicated by the yellow points (Fig. 2A,B). The ligature from the red point to the green point was the first incision on the top surface of the clavicle (Fig. 2A,B). Usually, the incision was approximately 20 mm long. Another 2-cm skin incision was made over the coracoid process (Fig. 2A), and the base of the coracoid process was exposed.

Usually, we can reduce the joint by pushing the elbow upward and maintaining the reduction in the supine position. Reduction of the dislocated AC joint can be confirmed by palpating the affected AC joint and comparing it with the normal AC joint. Furthermore, anteroposterior and axillary radiographic views by the C arm can confirm the anatomical reduction of the AC joint. If the reduction is inadequate, we can use large pointed reduction forceps placed on the coracoid process and the clavicle to assist in holding the reduction. The final AC joint reduction can be checked with the C arm to avoid overcorrection.

**Figure 4:**
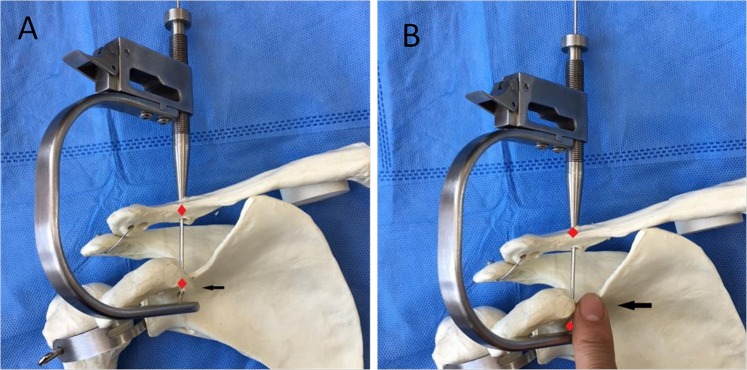
The placement of the conoid tunnels is determined on the distal clavicle and coracoid base in place of the conoid ligament. (**A**) The tip of the guider’s pin sleeve is centered on the conoid clavicular tunnel placement, as indicated by the red point (Fig. 2). The tip of the CC guider’s drill stop is placed beneath the medio-lateral coracoid base. The tip should touch the scapula. The medial borders of both the CC guider’s drill stop and coracoid base should be at the same level in the sagittal plane, as indicated by the black arrows. (**B**) The performer uses his index finger to touch the medial borders of both the CC guider’s drill stop and coracoid base to make them at the same level in the sagittal plane, as indicated by the black arrows. A 2-mm guide pin is inserted into the guider’s pin sleeve and advanced through the clavicle and coracoid, and its tip is captured by the drill stop of the CC guider at the base of the coracoid, as indicated by the red points.

**Figure 5:**
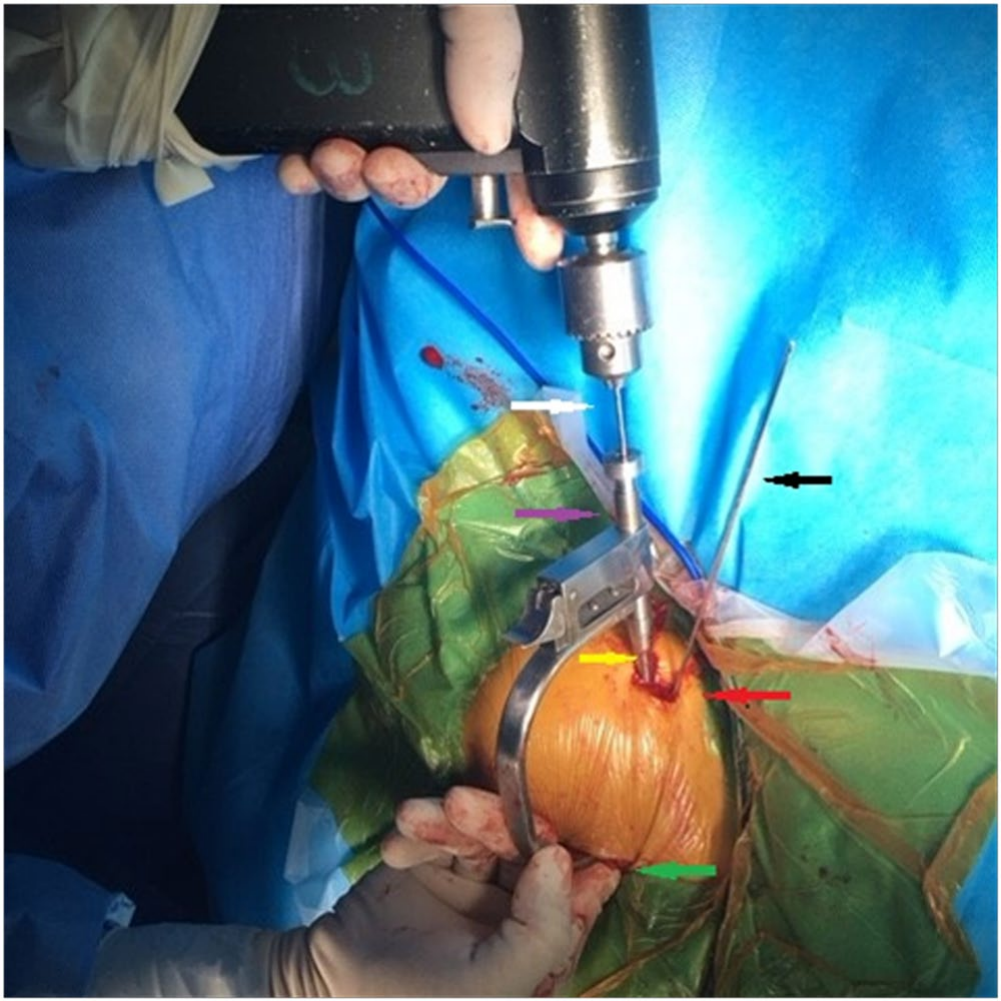
The placements of the tunnels are determined on the distal clavicle and conoid base during the operation. The CC guider is fixed in the two incisions. The tip of the guider’s pin sleeve is passed through the clavicular incision over the superior clavicle, as indicated by the yellow arrow. The tip of the CC guider’s drill stop should be placed across the coracoid incision beneath the medio-lateral coracoid base, as indicated by the green arrow. The placement of the conoid tunnels is determined on the distal clavicle and coracoid base by a 2-mm guide pin, as indicated by the black and red arrows. Another 2-mm guide pin is inserted into the guider’s pin sleeve and advanced through the clavicle and coracoid in place of the trapezoid ligament, as indicated by the white, purple and yellow arrows. The position of the CC guider’s drill stop in relation to the coracoid is confirmed by palpation of the performer’s index finger, as indicated by the green arrow. The guider’s pin sleeve is indicated by the yellow arrow, and the guider’s drill sleeve is indicated by the purple arrow.

Then, the CC guider was inserted into the two incisions. First, conoid clavicular and coracoid tunnels were made in the distal clavicle and coracoidal base to secure a TightRope system instead of the conoid ligament. The tip of the guider’s pin sleeve was passed through the incision over the superior clavicle. Its tip was centered at the placement of the conoid clavicular tunnel, as indicated by the red points in Figs 2 and 4. The tip of the CC guider’s drill stop was placed beneath the medio-lateral coracoid base (Fig. 4A). Its tip should touch the scapula (Fig. 4A). The medial borders of both the CC guider’s drill stop and the coracoid base were at the same levels in the sagittal plane (Fig. 4A). The position of the CC guider’s drill stop in relation to the coracoid base was confirmed by palpation with the surgeon’s index finger (Fig. 4B). Then, a power drill with a 2-mm tip guide pin was inserted into the guider’s pin sleeve and advanced through the clavicle and coracoids (Figs 4, 5). The tip of the guide pin was captured by the drill stop of the CC guider at the base of the coracoids (Fig. 4).

**Figure 6:**
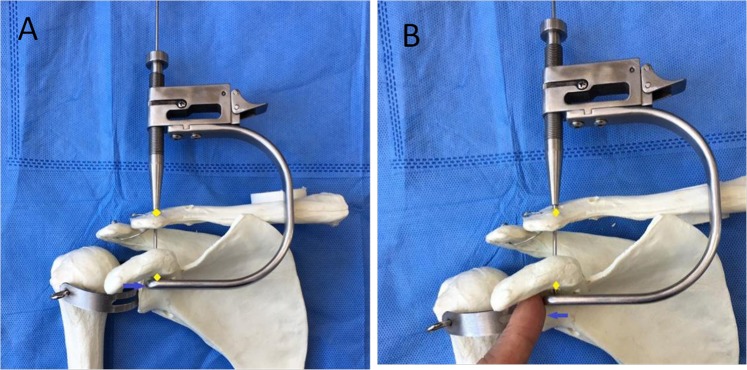
The placement of the trapezoid tunnels on the distal clavicle and coracoid base in place of the trapezoid ligament. (**A**) The tip of the guider’s pin sleeve is centered on the trapezoid clavicular tunnel placement, as indicated by the yellow point (Fig. 2). The tip of the CC guider’s drill stop is placed beneath the latero-medial coracoid base. It should touch the scapula. The lateral borders of both the CC guider’s drill stop and coracoid base are at the same level in the sagittal plane, as indicated by the blue arrows. (**B**) The performer uses his index finger to touch the lateral borders of both the CC guider’s drill stop and coracoid base to make them at the same level in the sagittal plane, as indicated by blue arrows. A 2-mm guide pin is inserted into the guider’s pin sleeve and advanced through the clavicle and coracoid, and its tip is captured by the drill stop of the CC guider at the base of the coracoid, as indicated by the yellow points.

**Figure 7:**
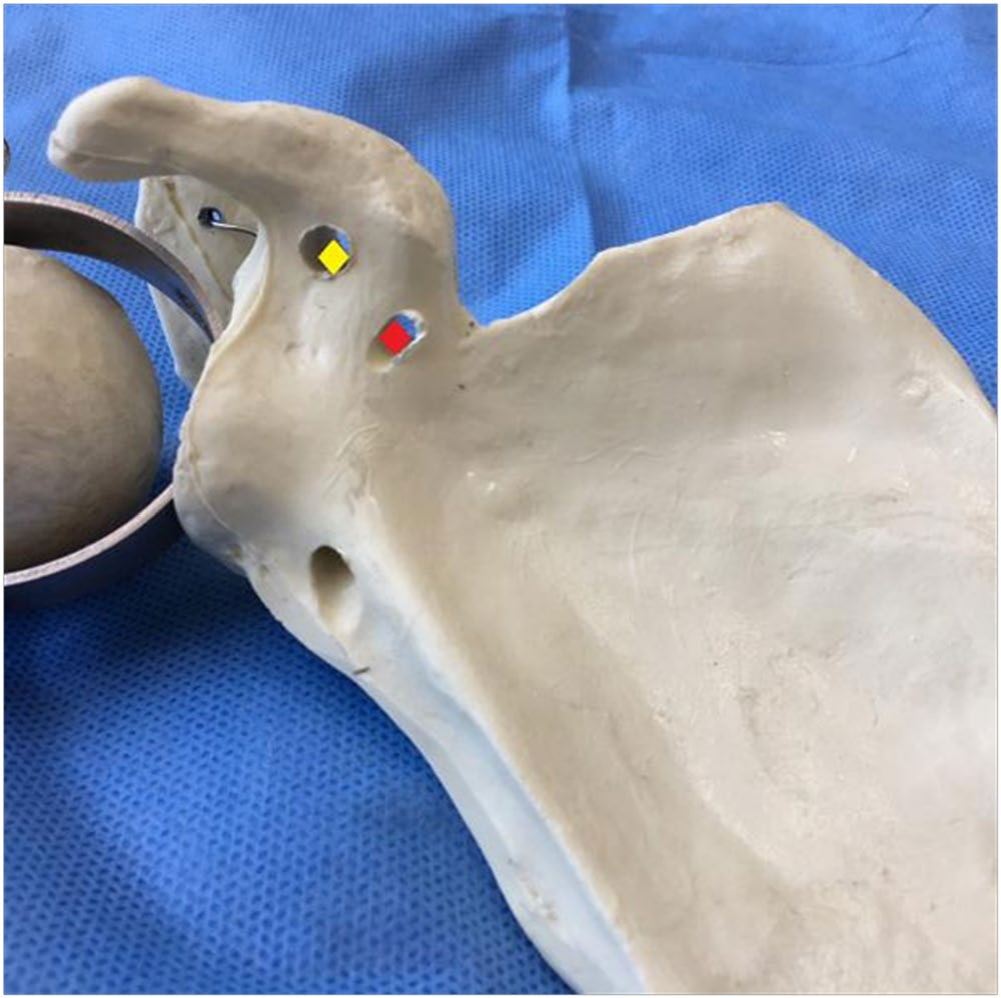
The conoid and trapezoid coracoid tunnel placements are marked beneath the coracoid base. The conoid coracoid tunnel placement is at the posterior aspect of the coracoid base, 5 mm lateral to the medial border, as indicated by red points. The trapezoid coracoid tunnel placement is 5 mm medial to the lateral border of the coracoid, as indicated by the yellow point. There is a >10-mm bony bridge between the two tips.

**Figure 8:**
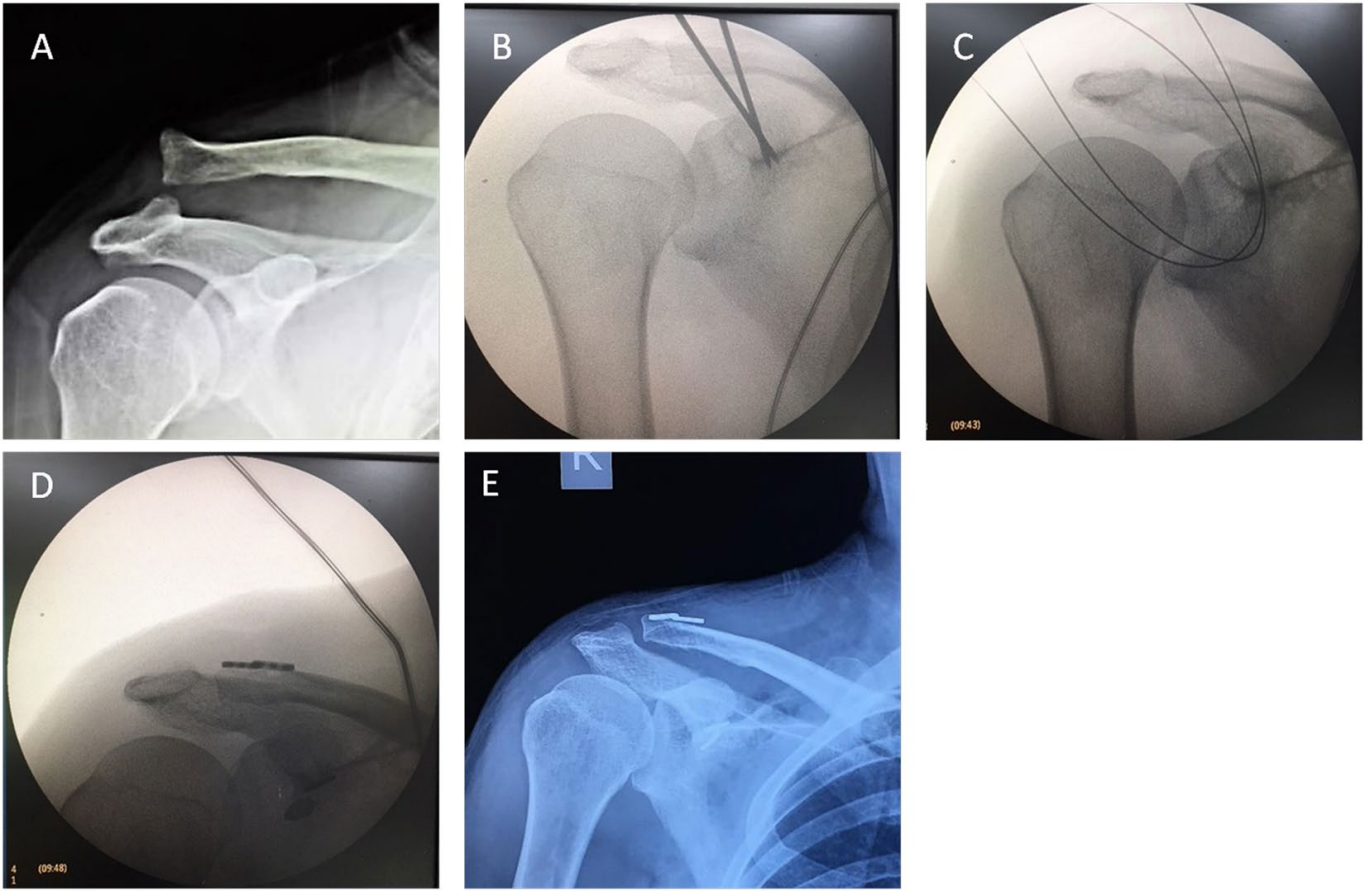
Pre- and post-operative radiographs of a right AC joint dislocation. (**A**) Preoperative X-ray of the right shoulder of a 46-year-old male patient who sustained a Neer type III AC joint dislocation after a fall. His coracoclavicular distance measured 29 mm, and his Constant score was 42. (**B**) Operative X-ray showing the 2 guide pins crossing from the clavicle to the coracoid base in place of the conoid and trapezoid ligaments. (**C**) Operative X-ray showing the 2 suture-passing wires cross through the clavicular and coracoid tunnels in place of the conoid and trapezoid ligaments. (**D**) Operative X-ray showing full reduction in the AC joint with the 2 TightRope systems reconstructing the trapezoid and conoid ligaments. (**E**) Post-operative X-ray of the right, dominant shoulder at the final follow-up 18 months after surgery demonstrating anatomical reduction of the AC joint with a coracoclavicular distance of 8 mm in both shoulders. His Constant score was 100.

Second, in the distal clavicle and conoidal base, trapezoid clavicular and coracoid tunnels were created to fix a TightRope system instead of the trapezoid ligament. The tip of the guider’s pin sleeve was centered at the placement of the trapezoid clavicular tunnel (Figs 1, 6). The tip of the CC guider’s drill stop was placed beneath the latero-medial coracoid base (Fig. 6A), and the tip touched the scapula (Fig. 6A). The lateral borders of both the CC guider’s drill stop and the coracoid base were at the same level in the sagittal plane (Fig. 6A). The position of the CC guider’s drill stop in relation to the coracoid was confirmed by palpation with the surgeon’s index finger (Fig. 6B). Then, another 2-mm drill tip guide pin was inserted into the guider’s pin sleeve and advanced through the clavicle and the coracoids (Figs 5, 6). At this time, the surgeon touched the tips of the two guide pins via the index finger to ensure that one guide pin tip was located at the posterior aspect of the coracoid base 5 mm lateral to the medial border. Another guide pin tip was placed 5 mm medial to the lateral border of the coracoid, and a >10-mm bony bridge existed between the two tips (Fig. 7). If the position was incorrect, the guide pin was reinserted. Additionally, the position of the pins in relation to the coracoid was assessed radiographically (Fig. 8B).

**Figure 9:**
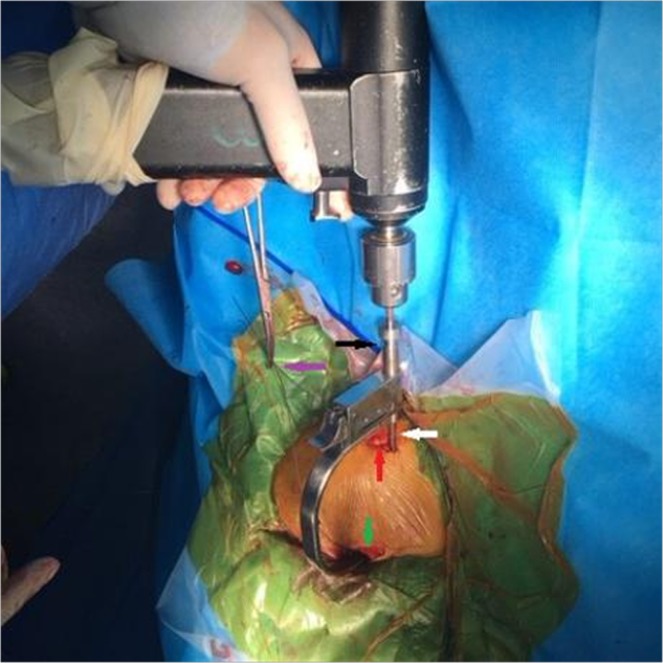
The 4-mm-diameter cannulated drill bit is advanced over the guide pin and through the clavicle and coracoid during the operation. Using a power drill, the 4-mm-diameter cannulated drill bit is inserted into the guider’s drill sleeve and is advanced over the pin and through the clavicle and coracoid in place of the conoid ligament, as indicated by the white arrow. The cannulated drill beyond the coracoid is stopped by the CC guider’s drill stop, as indicated by the green arrow. The clavicular incision is indicated by the red arrow. Another suture-passing wire, which has been pulled through the trapezoid tunnels from the clavicle to the coracoid base, is fixed by a hemostatic forceps, as indicated by the purple arrow.

**Figure 10:**
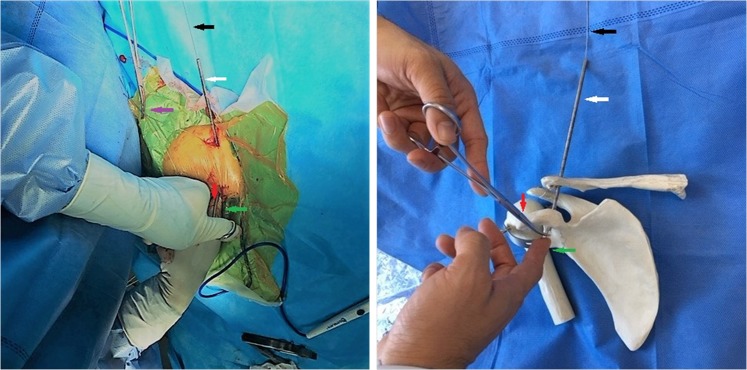
The suture-passing wire is advanced through the tunnels from the clavicle to the coracoid base during the operation. The guide pin is removed, and the cannulated drill bit is left *in situ*. A suture-passing wire was advanced down through the cannulated drill bit. The tip of the suture-passing wire is located under the coracoid by the performer’s index finger, and it is then grasped with a hemostatic forceps, as indicated by the green arrow. The suture-passing wire is indicated by the black arrow, the cannulated drill bit is indicated by the white arrow, and the hemostatic forceps is indicated by the red arrow. Another suture-passing wire, which has been pulled through the trapezoid tunnels from the clavicle to the coracoid base, is fixed by a hemostatic forceps, as indicated by the purple arrow.

When the correct positions of the guide pins were ensured, the guider’s pin sleeve was removed, and the guider’s drill sleeve was left *in situ* (Fig. 1B). Using a power drill, the performer inserted the 4-mm-diameter cannulated drill bit into the guider’s drill sleeve with the guide pin inside the cannulated drill bit, which was advanced along the pin and through the clavicle and the coracoid (Figs 1B, 9). The cannulated drill beyond the coracoid was stopped by the CC guider’s drill stop (Fig. 1B). The guide pin was removed, and the cannulated drill bit was left *in situ* (Fig. 10).

A suture-passing wire was advanced down through the cannulated drill bit. The suture-passing wire tip was located under the coracoid by the performer’s index finger, and it was then grasped with hemostatic forceps (Fig. 10). One of the two white traction sutures from the oblong button of the TightRope system was inserted through the suture-passing wire.

The suture-passing wire was pulled to retrieve the white traction suture from the coracoid incision to the clavicle incision. One traction suture was pulled to flip the oblong button into a vertical position suitable for advancement through the bone tunnels. The oblong button was advanced from the coracoid to the clavicle. The other white traction suture was pulled out when the oblong button emerged from the bone tunnel. Each oblong button suture was pulled independently to flip the button onto the clavicle surface.

The TightRope suture tails were pulled to advance the round button under the coracoid until the position of the clavicle reduction was noted to be satisfactory under direct visualization. First, the medial TightRope was tightened. The sutures were tied over the TightRope top using a surgeon’s knot and four additional half-hitches. This step completed the AC joint reduction and stabilization. Because suture tails were tied under the coracoid rather than on the surface of the clavicle, the knot stack could not be detected or touched from the skin. There are several advantages as a result of the insertion of the TightRope extending from the coracoid to the clavicle: 1. Because the suture tails were tied under the coracoid rather than on the surface of the clavicle, the knot stack could not be detected or touched from the skin. This avoided irritation and reduced foreign body reactions and infections. 2. The round button on the surface of the coracoid base can withstand more force, making failure less likely. 3. With the help of the knot transmitter, the TightRope can be easily tightened. Any remaining white traction sutures were removed by cutting and pulling them out of the buttons.

The fascia was closed in a full-thickness manner over the clavicle using several No. 1 absorbable sutures. Meticulous fascial closure is critical for restoring deltoid and trapezius function and for mediate hardware coverage by soft tissue. No. 2–0 absorbable sutures were used for dermal closure, and running No. 3-0 absorbable monofilament subcuticular sutures were used to close the skin.

Patients were asked to use shoulder immobilizers for 3 weeks, keeping them on except for when washing and conducting exercises to improve elbow extension and flexion. Patients were only allowed movement below shoulder height for the first 6 weeks, after which full movement was permitted. Patients were instructed to avoid any heavy resistance work for 3 months postoperatively.

### Statistical analysis

Paired t-tests were used to compare data SPSS, version 25.0 (https://www.ibm.com/cn-zh/). *p* values < 0.05 was the significance threshold.

## Results

Patients in this study resumed normal work within 7.25 weeks on average (range: 6–8 weeks), and did not suffer any significant complications. No additional surgeries were needed in these patients to treat recurrent episodes of AC joint dislocation. In all patients, the radiographic assessment conducted upon final follow-up indicated complete AC joint reduction. There was no significant difference between the CC distance on operative and contralateral in all patients (10.13 vs. 10.14 mm) (p > 0.05), with the metal buttons remaining properly positioned. The detailed patient information and results are shown in Table 1.

## Discussion

The ligamentous stabilizers of the AC joint consist of the AC joint and conoid and trapezoid ligaments^14^. The orientation and function of each ligament is different, providing AC joint stability^15,16^. The trapezoid and conoid constitute the CC ligaments^13^. Biomechanical studies in human cadaver specimens showed that the CC ligaments, primarily the conoid ligament, mainly constrain larger amounts of displacement and induced load, while the AC joint ligament mainly constrains lesser amounts of displacement and induced loads^10,17^. The CC ligaments are the primary stabilizers of the AC joint^12,13,16,17^.

While each ligament makes a different contribution to AC joint stability the primary current surgical techniques to treat AC joint dislocation rely upon CC interval fixation, thereby restoring CC ligaments without addressing the AC ligament^18^. The reason may be that we mainly deal with acute unstable ACJ injuries. When the TightRope system is used to restore the CC space, the torn ends of the ligaments are realigned, and they still have healing potential. Moreover, the double-row TightRope system has greater biomechanical strength considering both vertical or horizontal stability than native CC ligaments^13^. Therefore, repairing the CC ligaments is equivalent to transforming high-grade acromioclavicular dislocation into a low-grade injury, which can be treated conservatively.

In light of these findings, in 40 fresh-frozen cadaveric shoulders, reconstruction of the trapezoid and conoid ligaments was performed by placing two TightRope systems through individual tunnels from the clavicle to the coracoid^13^. Biomechanical measurements were performed in the shoulders and showed that this method withstands much higher forces than the native ligament complex^13^.

This is a stable and functional anatomic reconstruction procedure^13^. Our clinical findings were in accordance with these biomechanical findings. Via the new CC guider, we anatomically reconstructed the trapezoid and conoid of the CC ligament using 2 TightRope systems to manage acute AC joint dislocation.

In this procedure, the key point of the CC ligament reconstruction is to select the optimal clavicular and coracoid tunnel placements to install 2 TightRope systems in place of the trapezoid and conoid^13^. The new CC guider helps to confirm the tunnel placements, especially the coracoid tunnel placements beneath the coracoid.

First, we determined the optimal clavicular tunnel placements on top of the clavicle according to the cadaver study^13^. The trapezoid and conoid ligaments arise from the trapezoid and conoid tuberosity beneath the clavicle, respectively^19^. The trapezoid tubercle is positioned in the middle of the posterior third of the clavicle^19^, whereas the conoid tubercle is positioned in the posterior third of the clavicle^19^. In this study, the placement of the conoid clavicular tunnel was next to the conoid tubercle, i.e., the conoid insertion on the clavicle, and the placement of the trapezoid was at the mid-point between the clavicular distal end and the conoid tubercle (Fig. 2). Because the clavicle can be easily palpated from the body surface, confirming the optimal clavicular tunnel placements on top of the clavicle is easy (Fig. 2).

The conoid and trapezoid coracoid tunnel placements are selected beneath the coracoid base as shown in the aforementioned cadaver study^13^. The conoid ligament inserts into the medio-lateral coracoid base posteromedial to the trapezoid ligament, and the trapezoid coracoid insertion is at the latero-medial coracoid base^19^. Therefore, the conoid coracoid tunnel placement should be at the posterior aspect of the coracoid base 5 mm lateral to the medial border. The trapezoid coracoid tunnel placement should be 10 mm anterior to the conoidal tunnel and 5 mm medial to the lateral border of the coracoid, leaving a bony bridge between tunnels of at least 10 mm^13^. It is very difficult to determine the conoidal tunnel placements without arthroscopic assistance.

However, the CC guider can facilitate accurate coracoid tunnel placements beneath the coracoid. The centering device of the CC guider helps to center the guide pin accurately on the tip of the CC guider’s drill stop. The tip of the CC guider’s drill stop is semi-circular. Its diameter is 10 mm. The pin or cannulated drill, which passes through the CC guider’s pin sleeve or the CC guider’s drill sleeve, points accurately to the center of the semi-circular tip of the CC guider’s drill stop. The tip of the pin (or cannulated drill) is 5 mm from the border of the semi-circular tip of the CC guider’s drill stop. As long as the border of the semi-circular tip of the CC guider’s drill stop is not beyond the borders of the coracoid base, the coracoid tunnel placements are at least 5 mm from the borders of the coracoid base. Therefore, the performer can confirm the conoid coracoid tunnel placement as long as the medial borders of both the CC guider’s drill stop and coracoid base are at the same levels in the sagittal plane, which can be determined by palpation with the index finger (Fig. 4). Additionally, the performer can confirm the trapezoid coracoid tunnel placement as long as the lateral borders of both the CC guider’s drill stop and coracoid base are at the same level in the sagittal plane, which can be determined by palpation with the index finger (Fig. 6).

The drill stop of the CC guider is able to prevent the guide pin (or cannulated drill) from advancing too deep. Therefore, the CC guider is effective in preventing damage to the brachial plexus and axillary artery and vein. With the CC guider, the TightRope system can be safely and accurately used to fix the AC dislocation without arthroscopic assistance. This provides a simple, safe, effective, reproducible, and minimally invasive technique for acute AC joint stabilization that enables a rapid return to activity, leaves minimal scarring, and does not require hardware removal.

Acute AC joint reconstruction with the TightRope system (double-button system) is usually performed via an arthroscopic approach^18,20–22^. Excellent functional outcomes have been reported after arthroscopic AC joint reconstruction, so some authors recommend this procedure in any acute AC joint reconstruction^21^. The theoretical advantage of the arthroscopic approach is a better cosmetic result^5,20,21^. In the present study, we describe a new CC guider with which we can effectively restore the AC joint and CC ligament complex anatomy. A better cosmetic outcome can also be obtained. This technique is clinically easily performed with the CC guide. It requires less dissection and decreases the surgeon’s proximity to important neurovascular structures. The arthroscopic techniques require at least 3 incisions: one posterior portal, one anterolateral portal, and one mini-open incision on top of the clavicula^5,21^. Although the arthroscopic portals are small, they are usually relatively deep, and the coracoid base is highly exposed to enable arthroscope manipulation. We simply create two minimal incisions so that the CC guider can be directly positioned to clamp both the clavicle and coracoid. The procedure is fast and relatively simple. Through 2 small, 20-mm skin incisions made over the top of the clavicle and over the coracoid process, four bone tunnels can be drilled, with minimal damage to the soft tissues surrounding the CC ligaments, while accurate tunnel placement can be confirmed beneath the coracoid via palpation between the CC guider and coracoid. Our results are similar to those with arthroscopic procedures. Late removal of hardware was not routinely required, and the scars were cosmetically acceptable. No significant complications occurred. The patients had high functional scores and were happy with both the functional and cosmetic results. This technique repairs the CC ligament without the need to remove metalwork. In our series, the TightRope system was used in all cases. After a mean follow-up of 12 months, we did not record any bone erosion or loss of reduction secondary to implant failure. The short-term follow-up of 16 recently operated patients revealed excellent radiological and clinical results, with no subluxations or dislocations of the acromioclavicular joint noted(Table 1). The presented procedure may also be performed via an arthroscopic technique. The medial fragment of the clavicle following insertion of the conoid and trapezoid ligament was stabilized by 2 TightRope systems. In addition, surgeons should be aware of the potential risks related to the neurovascular structures underneath the clavicle and medial to the coracoid. There is a risk of fracture in the lateral aspect of the coracoid when tunneling through its base if the CC guide is placed too far laterally. Anteroposterior and axillary radiographic views should be obtained intraoperatively to confirm adequate reduction in the horizontal and vertical directions. The correct placement of the coracoid tunnels and the correct distance between them are crucial to prevent tunnel coalition and coracoids fracture. To prevent the loss of reduction (a common complication), the round button should lie flat on the surface of the coracoid base without soft tissue incarceration. If there is soft tissue incarceration, with the necrosis and disappearance of soft tissue, the TightRope tension will decrease, and the reduction will be lost.

In summary, the anatomical repair of CC ligaments with 2 TightRope systems via the presented CC guider yielded satisfactory functional and radiographic results for acute type III and IV AC joint dislocation. With the CC guider, the performer can ensure accurate placement of the clavicular and conoidal tunnel for installing 2 TightRope systems in place of the trapezoid and conoid ligaments, which is of paramount importance. In addition, this technique is able to avoid damage to the brachial plexus and axillary artery and vein under the coracoid while ensuring an excellent cosmetic result. Therefore, we recommend this technique for all type IV injuries and for type III injuries in workers with labor-intensive jobs and athletes who place a high demand on their upper extremities, such as pitchers.

## References

[1] Shin SJ, Kim N-K (2015). Complications After Arthroscopic Coracoclavicular Reconstruction Using a Single Adjustable–Loop-Length Suspensory Fixation Device in Acute Acromioclavicular Joint Dislocation. Arthroscopy the Journal of Arthroscopic & Related Surgery.

[2] Davies, E. J., Fagg, J. A. & Stanley, D. Subacromial, supracoracoid dislocation of the acromioclavicular joint with ipsilateral clavicle fracture: a case report with review of the literature and classification. *Journal of the Royal Society of Medicine Cardiovascular Disease 5*,*7*(*2014-01-07*) **5**, 2054270414527281 (2014). 10.1177/2054270414527281 PMC4100230 25057405

[3] Liu X, Huangfu X, Zhao J (2015). Arthroscopic treatment of acute acromioclavicular joint dislocation by coracoclavicular ligament augmentation. Knee Surgery Sports Traumatology Arthroscopy.

[4] Gartsman GM, Combs AH, Davis PF, Tullos HS (1991). Arthroscopic acromioclavicular joint resection. An anatomical study. American Journal of Sports Medicine.

[5] Murena L (2009). Arthroscopic treatment of acute acromioclavicular joint dislocation with double flip button. Knee Surgery Sports Traumatology Arthroscopy.

[6] Vulliet, P., Hanneur, M. L., Cladiere, V., Loriaut, P. & Boyer, P. A comparison between two double-button endoscopically assisted surgical techniques for the treatment acute acromioclavicular dislocations. *Musculoskeletal Surgery*, 1–7 (2017). 10.1007/s12306-017-0501-0 28861851

[7] Xu J (2018). A retrospective comparative study of arthroscopic fixation in acute Rockwood type IV acromioclavicular joint dislocation: single versus double paired Endobutton technique. Bmc Musculoskeletal Disorders.

[8] Marco S, Mauro DC, Alessio Giai V, Francesco O (2015). All arthroscopic stabilization of acute acromioclavicular joint dislocation with fiberwire and endobutton system. Muscles Ligaments & Tendons Journal.

[9] Mazzocca AD (2006). A biomechanical evaluation of an anatomical coracoclavicular ligament reconstruction. American Journal of Sports Medicine.

[10] Yoo YS (2010). A Biomechanical Analysis of the Native Coracoclavicular Ligaments and Their Influence on a New Reconstruction Using a Coracoid Tunnel and Free Tendon Graft. Arthroscopy the Journal of Arthroscopic & Related Surgery.

[11] Takase K (2010). The coracoclavicular ligaments: an anatomic study. Surgical & Radiologic Anatomy Sra.

[12] Zhu NF, Rui BY, Zhang YL, Chen YF (2016). Anatomic study of coracoclavicular ligaments for reconstruction of acromioclavicular joint dislocations. Journal of Orthopaedic Science Official Journal of the Japanese Orthopaedic Association.

[13] Walz L, Salzmann GT (2008). The anatomic reconstruction of acromioclavicular joint dislocations using 2 TightRope devices: a biomechanical study. American Journal of Sports Medicine.

[14] Knut B (2014). Rotational and translational stability of different methods for direct acromioclavicular ligament repair in anatomic acromioclavicular joint reconstruction. American Journal of Sports Medicine.

[15] Le, H. M. *et al*. Biomechanical Comparison of Anatomic and Extra-Anatomic Reconstruction Techniques Using Local Grafts for Chronic Instability of the Acromioclavicular Joint. *Am J Sports Med* (2018). 10.1177/0363546518770603 29746150

[16] Costic RS, Labriola JE, Rodosky MW, Debski RE (2004). Biomechanical rationale for development of anatomical reconstructions of coracoclavicular ligaments after complete acromioclavicular joint dislocations. Am J Sports Med.

[17] Salzmann GM, Jochen P, Sandmann GH, Imhoff AB, Schöttle PB (2008). The coracoidal insertion of the coracoclavicular ligaments: an anatomic study. American Journal of Sports Medicine.

[18] Shin SJ, Campbell S, Scott J (2014). Simultaneous anatomic reconstruction of the acromioclavicular and coracoclavicular ligaments using a single tendon graft. Knee Surgery Sports Traumatology Arthroscopy Official Journal of the Esska.

[19] Rios CG, Arciero RA, Mazzocca AD (2007). Anatomy of the clavicle and coracoid process for reconstruction of the coracoclavicular ligaments. Am J Sports Med.

[20] Wolf EM, Pennington WT (2001). Arthroscopic reconstruction for acromioclavicular joint dislocation. Arthroscopy the Journal of Arthroscopic & Related Surgery.

[21] Acar MA (2015). Percutaneous double-button fixation method for treatment of acute type III acromioclavicular joint dislocation. Acta orthopaedica et traumatologica turcica.

[22] Pascal B, Jason O, Olivier G, Nicolas B, Yannick R (2010). All-arthroscopic Weaver-Dunn-Chuinard procedure with double-button fixation for chronic acromioclavicular joint dislocation. Arthroscopy: The Journal of Arthroscopic and Related Surgery.

